# Nano-drug delivery system for pancreatic cancer: A visualization and bibliometric analysis

**DOI:** 10.3389/fphar.2022.1025618

**Published:** 2022-10-18

**Authors:** Jie-Feng Zhao, Fei-Long Zou, Jin-Feng Zhu, Chao Huang, Fan-Qin Bu, Zheng-Ming Zhu, Rong-Fa Yuan

**Affiliations:** Department of General Surgery, The Second Affiliated Hospital of Nanchang University, Nanchang, Jiangxi, China

**Keywords:** pancreatic cancer, nano-drug delivery system, bibliometric analysis, CiteSpace, R-bibliometrix, VOSviewer

## Abstract

**Background:** Nano drug delivery system (NDDS) can significantly improve the delivery and efficacy of drugs against pancreatic cancer (PC) in many ways. The purpose of this study is to explore the related research fields of NDDS for PC from the perspective of bibliometrics.

**Methods:** Articles and reviews on NDDS for PC published between 2003 and 2022 were obtained from the Web of Science Core Collection. CiteSpace, VOSviewer, R-bibliometrix, and Microsoft Excel were comprehensively used for bibliometric and visual analysis.

**Results:** A total of 1329 papers on NDDS for PC were included. The number of papers showed an upward trend over the past 20 years. The United States contributed the most papers, followed by China, and India. Also, the United States had the highest number of total citations and H-index. The institution with the most papers was Chinese Acad Sci, which was also the most important in international institutional cooperation. Professors Couvreur P and Kazuoka K made great achievements in this field. *JOURNAL OF CONTROLLED RELEASE* published the most papers and was cited the most. The topics related to the tumor microenvironment such as “tumor microenvironment”, “tumor penetration”, “hypoxia”, “exosome”, and “autophagy”, PC treatment-related topics such as “immunotherapy”, “combination therapy”, “alternating magnetic field/magnetic hyperthermia”, and “ultrasound”, and gene therapy dominated by “siRNA” and “miRNA” were the research hotspots in the field of NDDS for PC.

**Conclusion:** This study systematically uncovered a holistic picture of the performance of NDDS for PC-related literature over the past 20 years. We provided scholars to understand key information in this field with the perspective of bibliometrics, which we believe may greatly facilitate future research in this field.

## Introduction

As an extremely malignant digestive system tumor, pancreatic cancer (PC) has atypical clinical symptoms (Strobel et al., 2019), progresses rapidly, and lacks sensitive biomarkers for early diagnosis (Siegel et al., 2019), resulting in more than 80% of PC patients being in advanced stage at the initial diagnosis and missing the opportunity of surgical resection (Xie et al., 2018). At present, the efficacy of commonly used chemotherapy schemes, such as FOLFIRINOX and gemcitabine plus nab-paclitaxel, is still limited (Kielaite-Gulla et al., 2022). The main reasons that hinder these chemotherapy drugs from significantly improving the prognosis of the patients are the biological complexity and heterogeneity of PC (Fan et al., 2017), the poor specificity of chemotherapy drugs, uneven distribution in the body, and side effects on normal tissues and organs (Peng et al., 2017), and chemotherapy resistance of PC (Stott et al., 2022). The clinical application of immunotherapy also faces challenges: first, the human immune system is difficult to accurately distinguish tumor cells from normal cells, resulting in “targeted extra tumor” toxicity (Yilmaz et al., 2020); Secondly, the complex immunosuppressive microenvironment of solid tumors prevents intravenous infusion of immune cells or cytokines from reaching the tumor site (Lu et al., 2021).

Therefore, finding an ideal biocompatible targeted drug delivery system to overcome the above difficulties has become a research hotspot (Lammers et al., 2012). Nanomaterial has a small volume, large surface area (Rai et al., 2021), high permeability, can be effectively combined with a variety of biological materials to improve their biocompatibility, and can effectively control drug release (Chen et al., 2016). Therefore, a nano-drug delivery system (NDDS) can protect encapsulated drugs from blood circulation degradation, target drug delivery, reduce systemic toxicity, improve drug solubility, and improve drug pharmacokinetics and therapeutic efficacy (Kaushik et al., 2022). NDDS works best in areas where many limitations exist with molecular targeted therapy, and a need exists to push treatment boundaries for PC (Kokkinos et al., 2020).

Currently, bibliometrics has become a very important methodology for scholars to effectively identify the latest progress in a certain research field, predict research hotspots, and evaluate the development trend of this field. In recent years, bibliometrics has been applied to the research field related to nanomaterials (Huang et al., 2015; Zhu et al., 2021; Huang et al., 2022). However, there is still a lack of bibliometric analysis of the application of nanomaterials in oncology in the face of the research trend of oncology, materials science, pharmacology, and other disciplines gradually interpenetrating. As far as we know, there was no bibliometric analysis of NDDS for PC at present. In this study, based on the Web of Science Core Collection (WOSCC) database, we used CiteSpace, VOSviewer, and R-Bibliometrix to conduct bibliometric and visual analysis on the number of publications, citations, and research trends of countries/regions, institutions, authors, and keywords in NDDS for PC related literature, and sorted out the research hotspots and predicted the development trends in this field.

## Methods

### Data sources and search strategies

**FIGURE 1:**
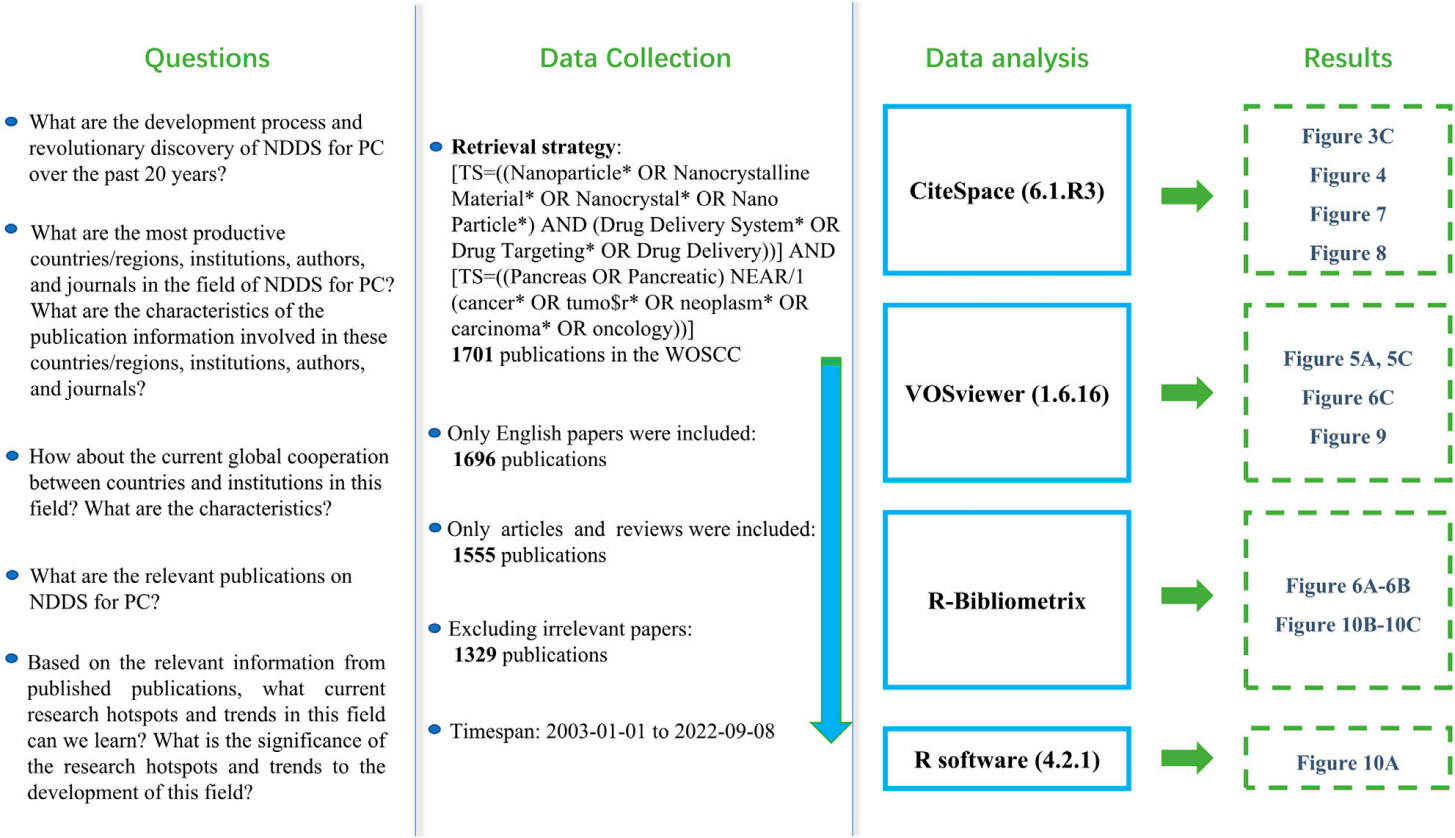
The research flow chart of this study.

We comprehensively searched publications related to NDDS for PC in the WOSCC database from 2003 to 2022 (as of September 8).Reasonable use of wildcards makes retrieval strategies more organized and scientific (Cheng et al., 2022a; Cheng et al., 2022b). The retrieval strategy of this study was as follows: [TS=((Nanoparticle* OR Nanocrystalline Material* OR Nanocrystal* OR Nano Particle*) AND (Drug Delivery System* OR Drug Targeting* OR Drug Delivery))] AND [TS=((Pancreas OR Pancreatic) NEAR/1 (cancer* OR tumo$r* OR neoplasm* OR carcinoma* OR oncology))]. Only English language publications were included; the data category was limited to “article” and “review”. After excluding the publications that meet the language and article type requirements, further, we evaluated the title and abstract articles to determine whether the literature meets the theme of NDDs for PC. For the uncertain literature, the full text was downloaded and evaluated in more detail. Figure 1 showed the research flow chart.

### Bibliometric analysis

CiteSpace (6.1.R3) was used to analyze the included literature, including co-citation analysis performed on countries/regions and institutions, dual-map overlay of citations, timeline view, co-cited references analysis, and references with the strongest citation burst.

VOSviewer (1.6.16) was used to visualize the co-citation network of authors and journals, and the co-occurrence of keywords. In the visual map, different nodes represented authors, journals, or keywords, etc; The node size indicated numbers or frequency; The thickness of the line represented the strength of the link; The colors of nodes represented different clusters or times.

R-Bibliometrix was used to analyze the theme evolution based on keywords over time, visualize the cooperation network between countries, and make a descriptive analysis of the publishing characteristics of journals. In addition, we used R software and R-Bibliometrix to generate the distribution map of high-frequency keywords over time.

Moreover, the scientometric online platform (https://bibliometric.com/) was used to conduct international cooperation networks between countries.

## Results

### Annual publications and trend

**FIGURE 2:**
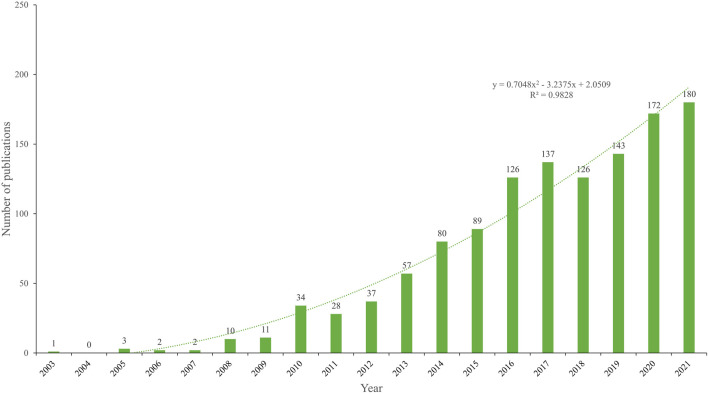
Number of annual publications regarding NDDS for PC from 2003 to 2021. Each bar shows the number of publications per year. The dotted line represents the curve fitting for this trend. Abbreviation: NDDS, Nano-drug Delivery System; PC, pancreatic cancer.

A total of 1329 publications regarding NDDS for PC were included. As shown in Figure 2, in general, since 2008, the annual number of publications has exceeded 10 and increased over time. The number of publications of NDDS for PC was the highest in 2021, although there was a slight decline in volatility in some years (2011 and 2018). 1329 publications had a total of 57,431 citations, with an average of 43.21 citations per paper, and an H-index of 102.

### Countries/regions

**FIGURE 3:**
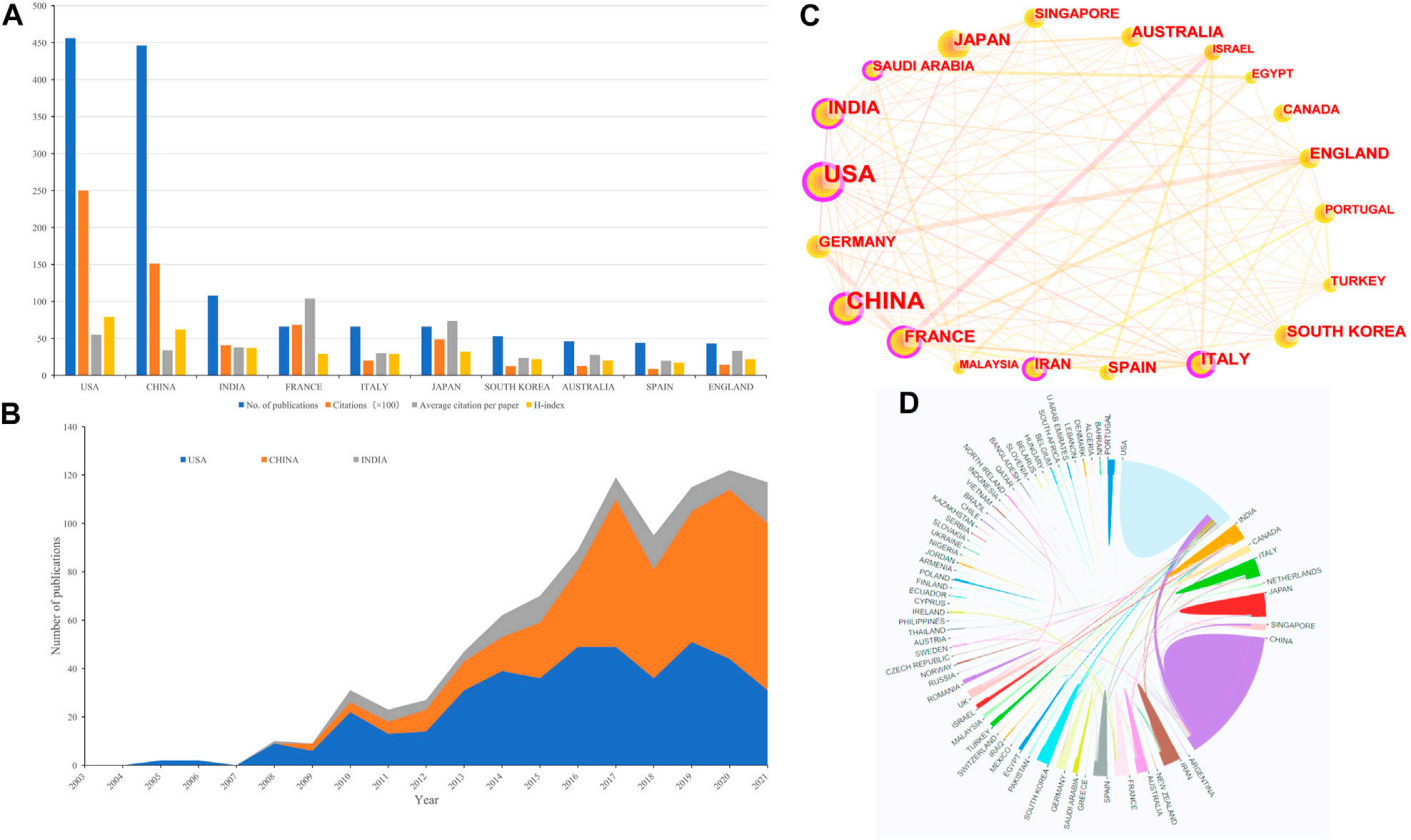
**(A)** The number of publications, total citations (×100), average citation per paper, and H-index of the 10 most productive countries/regions. **(B)** Annual output trend of the top 3 productive countries. **(C)** The country cooperation network generated by Citespace. Each node reprent a country, and the size of each node is proportional to the number of publications. The links between countries reflect co-occurrence relationships, while line thickness reflects the strength of cooperation. **(D)** The international cooperation networks between countries. Line thickness between countries reflects the intensity of the closeness.

A total of 71 countries/regions contributed to the publications included. The United States had the largest number of publications (*n* = 456, accounting for 34.312% of the total; 25,009 citations, with an average of 54.84 citations per paper, and an H-index of 79), followed by China (*n* = 446, accounting for 33.559%; 15,117 citations, with an average of 33.89 citations per paper, and an H-index of 62), and India (*n* = 108, accounting for 8.126%; 4,071 citations, with an average of 37.69 citations per paper, and an H-index of 37) (Figure 3A). Although the total number of publications in France ranked fourth, its total number of citations was second only to the United States and China, and its average number of citations was higher than that of the United States. Figure 3B summarized the annual output trend of the top 3 productive countries from 2003 to 2021. Figures 3C,D showed international cooperation among countries. Figure 3C was generated by CiteSpace: The thickness of the lines between countries showed the strength of cooperation. Among the top 20 countries with the most papers, the United States and China had the closest cooperation with other countries/regions; The United States, European countries (e.g., Italy and France), and Asian countries (e.g., China, India, and Saudi Arabia) were still the most important part of the national cooperation map, and extensive cooperative relations have been established between them, but the cooperation of other developing countries was still weak. In addition, cooperation in neighboring regions (e.g., the United States and Canada; China and Japan, South Korea), intra-continental cooperation (e.g., Germany and Spain, England, and Italy), and inter-continental cooperation (e.g., China and the United States) were also observed.

### Institutions

**FIGURE 4:**
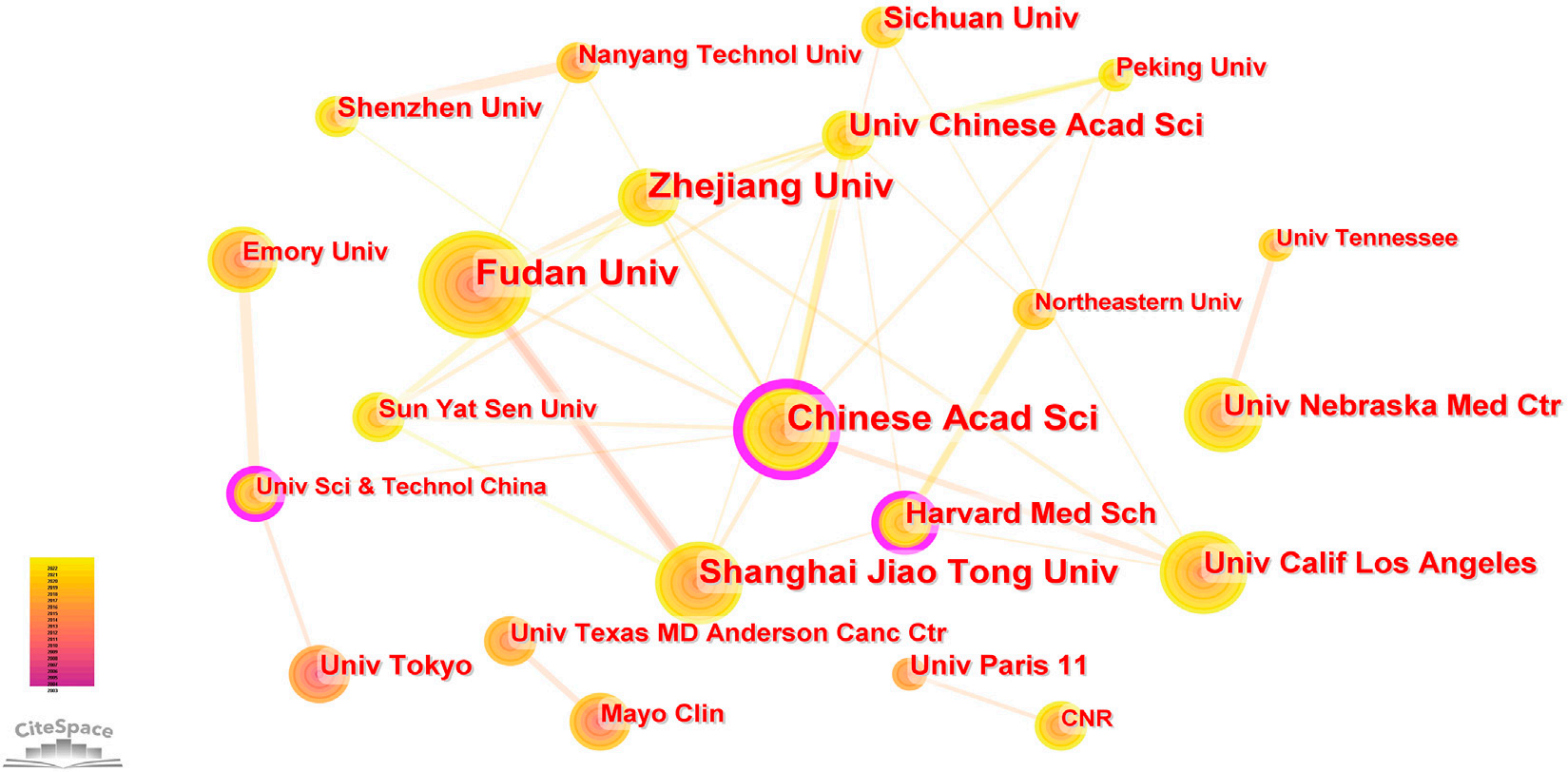
The cooperation network of institutions generated by Citespace. In the visualization map, one node represents an institution, and its size is proportional to the number of publications. The links between nodes represent the strength of cooperation.

A total of 1,600 institutions worldwide contributed to the 1329 publications related to NDDS for PC. CiteSpace generated a cooperation network of institutions, as shown in Figure 4. The top 5 institutions with the most papers were Chinese Acad Sci, Fudan Univ, Zhejiang Univ, Shanghai Jiao Tong Univ, and Univ Chinese Acad Sci. The betweenness centrality (BC) value was an index to evaluate the importance of nodes in a collaborative network, and a BC value >0.1 was considered a vital node (Cheng et al., 2022b). Among the top 22 institutions with the most publications, institutions with BC values greater than 0.1 included Chinese Acad Sci (0.27), Harvard Med Sch (0.11), and Univ Sci and Technol China (0.11). Among these 22 institutions, China had 10 institutions, and the United States had 8 institutions (Table 1).

**TABLE 1:** Ranking of the top 22 institutions with the most publications.

Rank	Institutions	Country	No. of publications	Centrality
1	Chinese Acad Sci	Peoples R China	50	0.27
2	Fudan Univ	Peoples R China	47	0.05
3	Zhejiang Univ	Peoples R China	47	0.05
4	Shanghai Jiao Tong Univ	Peoples R China	36	0.02
5	Univ Chinese Acad Sci	Peoples R China	33	0.1
6	Univ Calif Los Angeles	United States	25	0.04
7	Univ Nebraska Med Ctr	United States	24	0.04
8	Harvard Med Sch	United States	22	0.11
9	Sichuan Univ	Peoples R China	21	0.02
10	Shenzhen Univ	Peoples R China	18	0.07
11	Univ Paris 11	France	17	0.03
12	Univ Tokyo	Japan	16	0.07
13	Univ Texas MD Anderson Canc Ctr	United States	15	0.02
14	Emory Univ	United States	15	0.02
15	Sun Yat Sen Univ	Peoples R China	15	0
16	Nanyang Technol Univ	Singapore	14	0.08
17	Mayo Clin	United States	13	0.06
18	Peking Univ	Peoples R China	13	0.01
19	Univ Sci and Technol China	Peoples R China	11	0.11
20	Northeastern Univ	United States	11	0.03
21	CNR	Italy	11	0.02
22	Univ Tennessee	United States	11	0.01

### Authors and co-cited authors

**FIGURE 5:**
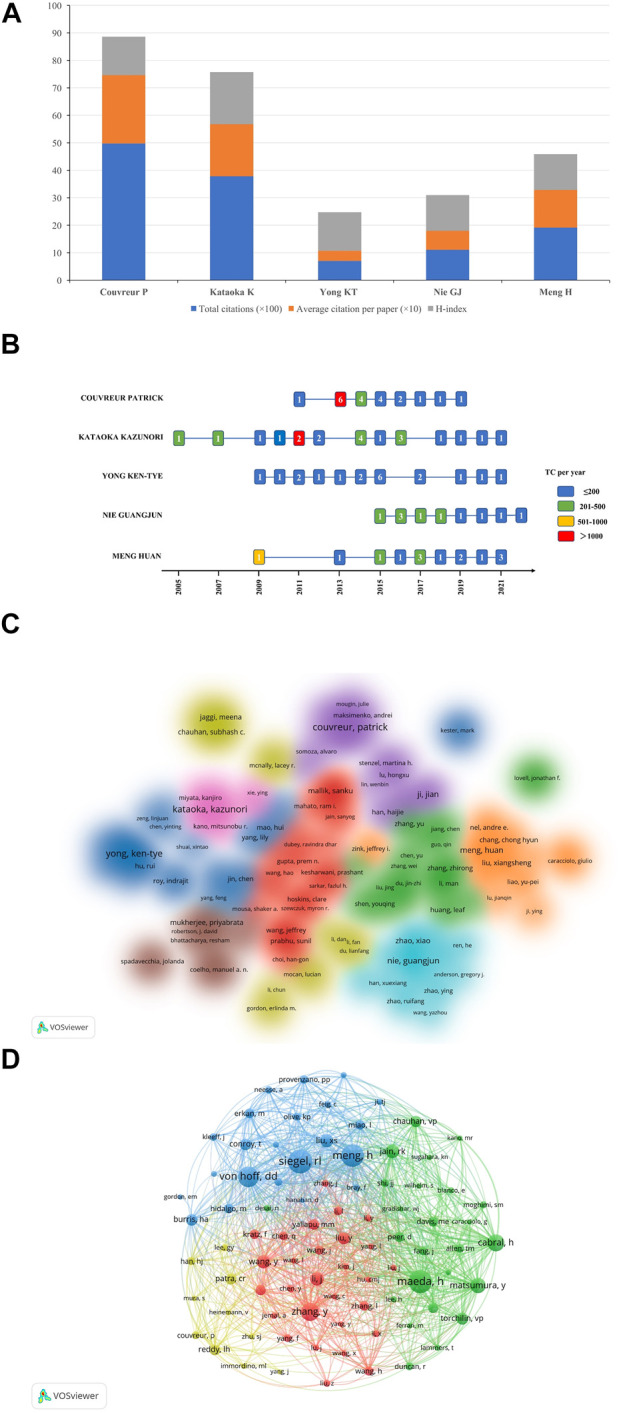
**(A)** The total citations (×100), average citation per paper (×10), and H-index of the top five most prolific authors. **(B)** The number of publications by the top five most prolific authors in different years and the total citations per year. Different color of each cell reflects different number of TC per year, and the number in the cell represents the number of publications each year. **(C)** Author co-authorship analysis by VOSviewer. One color reprents a cluster, and authors with close relationship are allocated to the same cluster. **(D)** Author co-citation analysis by VOSviewer. One node represents an author, and the lines between nodes represent the co-citation relationship. Abbreviation: TC, total citations.

In terms of an author analysis, the total citations (×100), average citation per paper (×10), and H-index of the top five most prolific authors were shown in Figure 5A. Couvreur P contributed the most publications, followed by Kataoka K and Yong KT. The publications of Couvreur P had the highest total and average citations (4976 and 248.8, respectively), and the H-index is 14; The total and average citations of publications by Kataoka K ranked second (3784 and 189.2 respectively), with the highest H-index (*n* = 19); Although the number of publications by Meng H ranked fifth, the total and average number of citations of which ranked third (1918 and 137 respectively). Figure 5B showed the number of publications by the top five most prolific authors in different years and the total citations per year: It could be seen that the total citations of the six papers by Couvreur P in 2013 exceeded 1000 (*n* = 4,306), the total citations of two papers by Kataoka K in 2011 exceeded 1000 (*n* = 1,870), and one article by Meng H in 2009 was cited 687 times in total. Figure 5C showed the cluster density map of author co-authorship analysis. Only 158 authors with more than 5 papers were included, forming a total of 9 author clusters. By analyzing the co-citation network of authors, 85 authors who have been cited more than 45 times were defined as key researchers (Figure 5D): The connection represented the cooperation between authors, and the size of the circle represented the number of citations. Total link strength (TLS) indicated the impact of authors’ published papers on other authors involved in the studies. Meng H had the greatest TLS (*n* = 2134), followed by Von Hof DD (*n* = 1597), and Maeda H (*n* = 1582).

### Journals

**FIGURE 6:**
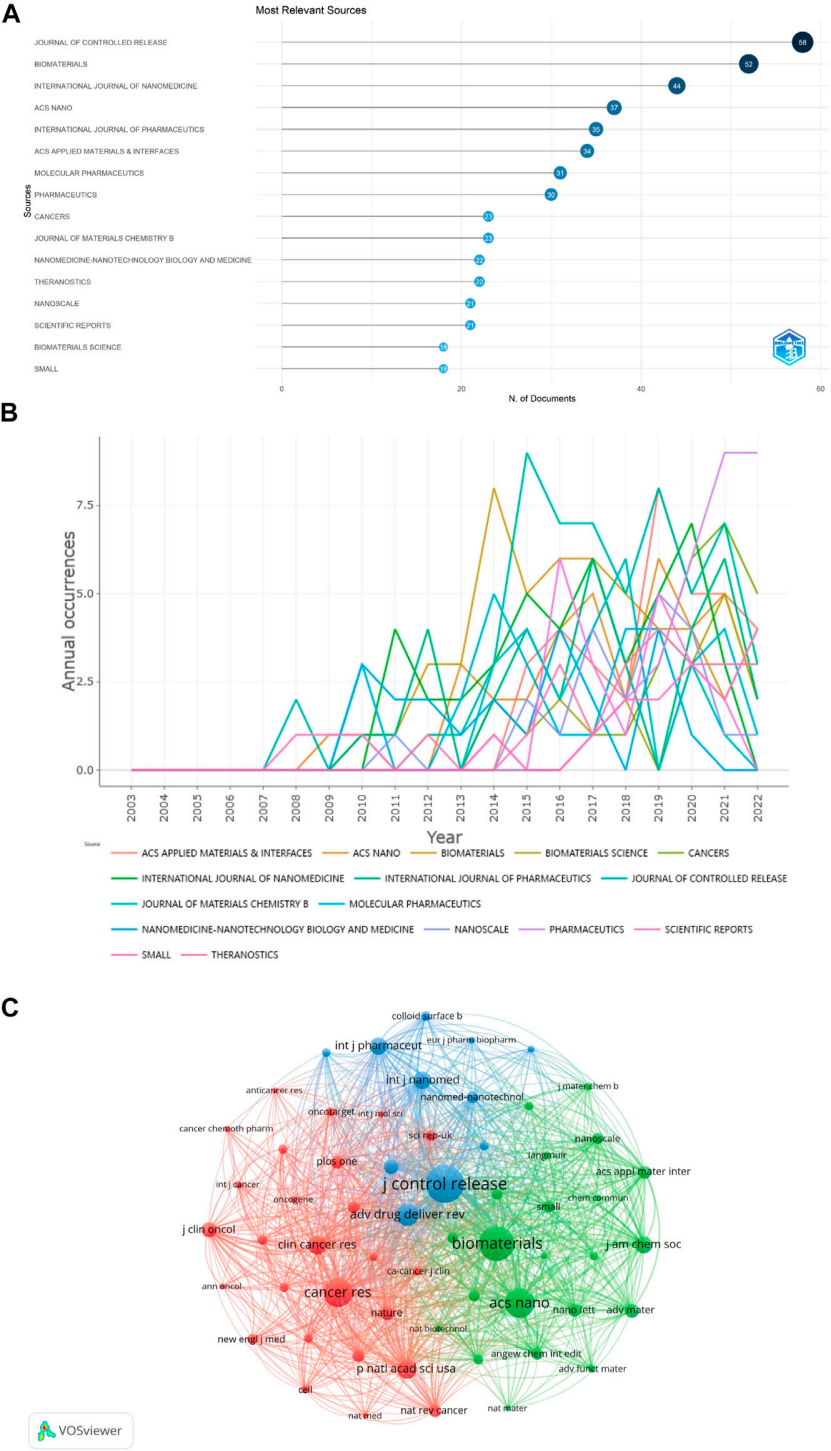
**(A)** The top 16 journals with the most publications generated by R-Bibliometrix. **(B)** Annual occurrences of the top 16 journals with the most publications generated by R-Bibliometrix. **(C)** The network visualization diagram of journal co-citation analysis generated by VOSviewer. One node represents one journal, and the area means the citation frequency. Sizes of the nodes are reflected with co-citations.

329 journals published 1329 publications regarding NDDS for PC, and 66 journals published more than 5 publications. Among the top 16 journals in terms of the number of papers, *JOURNAL OF CONTROLLED RELEASE* ranked first (58 papers), followed by *BIOMATERIALS* and *INTERNATIONAL JOURNAL OF NANOMEDICINE* (52 and 44 papers, respectively) (Table 2; Figure 6A). Among these 16 journals, 5 were published in the Netherlands, 5 were published in England, and 3 in the United States. The papers of *ACS NANO* had the highest total number of citations (*n* = 4,110), followed by *BIOMATERIALS* (*n* = 3,294), and *JOURNAL OF CONTROLLED RELEASE* (*n* = 2,577). According to the latest JCR division in 2021, 13 journals were in JCR Q1 and 3 journals were in JCR Q2, and *ACS NANO* was the journal with the highest impact factor. In addition, we also conducted the map of annual occurrences of the top 16 journals, to more specifically understand the changing trend of the number of publications of these journals in different years (Figure 6B). As shown in Figure 6C, the network visualization diagram of journal co-citation analysis was created by VOSviewer. Only visually cite journals at least 300 times. Among the 59 journals that met the standard, the top 5 journals commonly cited were *JOURNAL OF CONTROLLED RELEASE*, *BIOMATERIALS*, *ACS NANO*, *CANCER RESEARCH,* and *ADVANCED DRUG DELIVERY REVIEWS*.

**TABLE 2:** The top 16 journals with the most publications regarding NDDS for PC.

Rank	Journals	Publications	Citations	IF (2021)	JCR (2021)	Country
1	JOURNAL OF CONTROLLED RELEASE	58	2,577	11.467	Q1	Netherlands
2	BIOMATERIALS	52	3,294	15.304	Q1	Netherlands
3	INTERNATIONAL JOURNAL OF NANOMEDICINE	44	1,754	7.033	Q2	New Zealand
4	ACS NANO	37	4,110	18.027	Q1	United States
5	INTERNATIONAL JOURNAL OF PHARMACEUTICS	35	1,023	6.51	Q1	Netherlands
6	ACS APPLIED MATERIALS INTERFACES	34	870	10.383	Q1	United States
7	MOLECULAR PHARMACEUTICS	31	1,141	5.364	Q1	United States
8	PHARMACEUTICS	30	252	6.525	Q1	Switzerland
9	JOURNAL OF MATERIALS CHEMISTRY B	23	490	7.571	Q1	England
10	CANCERS	23	431	6.575	Q1	Switzerland
11	NANOMEDICINE NANOTECHNOLOGY BIOLOGY AND MEDICINE	22	1,066	6.458	Q2	Netherlands
12	THERANOSTICS	22	740	11.6	Q1	Australia
13	NANOSCALE	21	520	8.307	Q1	England
14	SCIENTIFIC REPORTS	21	494	4.996	Q2	England
15	SMALL	18	1,162	15.153	Q1	Germany
16	BIOMATERIALS SCIENCE	18	319	7.59	Q1	England

Abbreviations: PC, pancreatic cancer; NDDS, Nano-drug Delivery System ; IF, impact factor; JCR, Journal Citation Reports.

### Dual-map overlays of nano drug delivery system for pancreatic cancer

**FIGURE 7:**
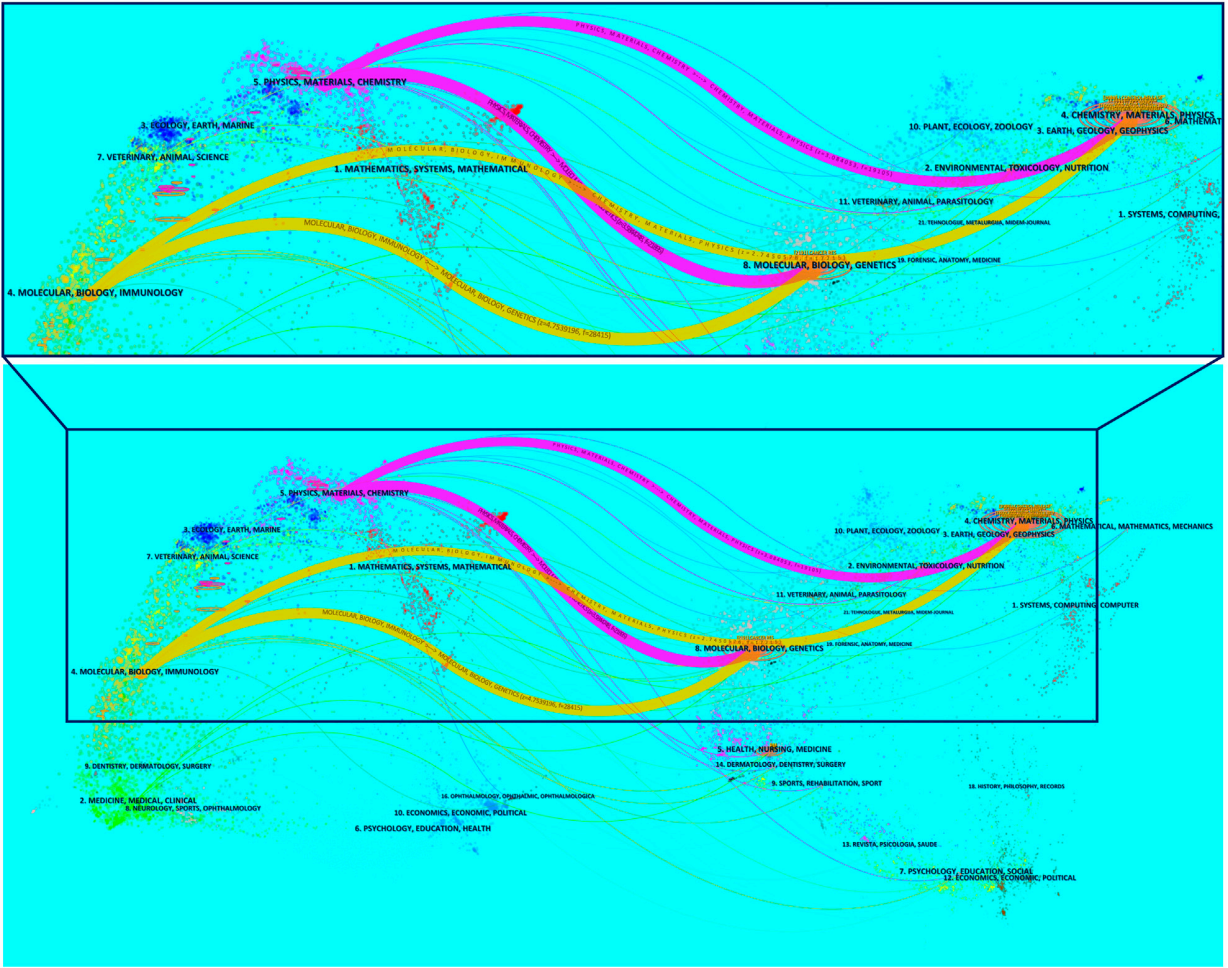
The dual-map overlay of journals contributed to publications regarding NDDS for PC from 2003 to 2022. The left side was the citing journal, the right side was the cited journal, and the colored line path represented the citation relationship. In order to display the local area more precisely, the local area is magnified (the area enclosed by the box). Abbreviation: NDDS, Nano-drug Delivery System; PC, pancreatic cancer.

The superposition of dual-map overlays revealed the overall scientific contribution. The left side was the citing journal, the right side was the cited journal, and the colored line path represented the citation relationship, indicating the citation trajectory and knowledge flow of knowledge (Chen and Leydesdorff, 2014). The result indicated that the citing papers regarding NDDS for PC were mainly focused on journals in the field of molecular, biology, immunology, and physics, materials, chemistry, whereas most of the cited articles were published in journals in the field of molecular, biology, genetics, and chemistry, materials, physics (Figure 7).

### Co-cited references and the strongest citation burst

**FIGURE 8:**
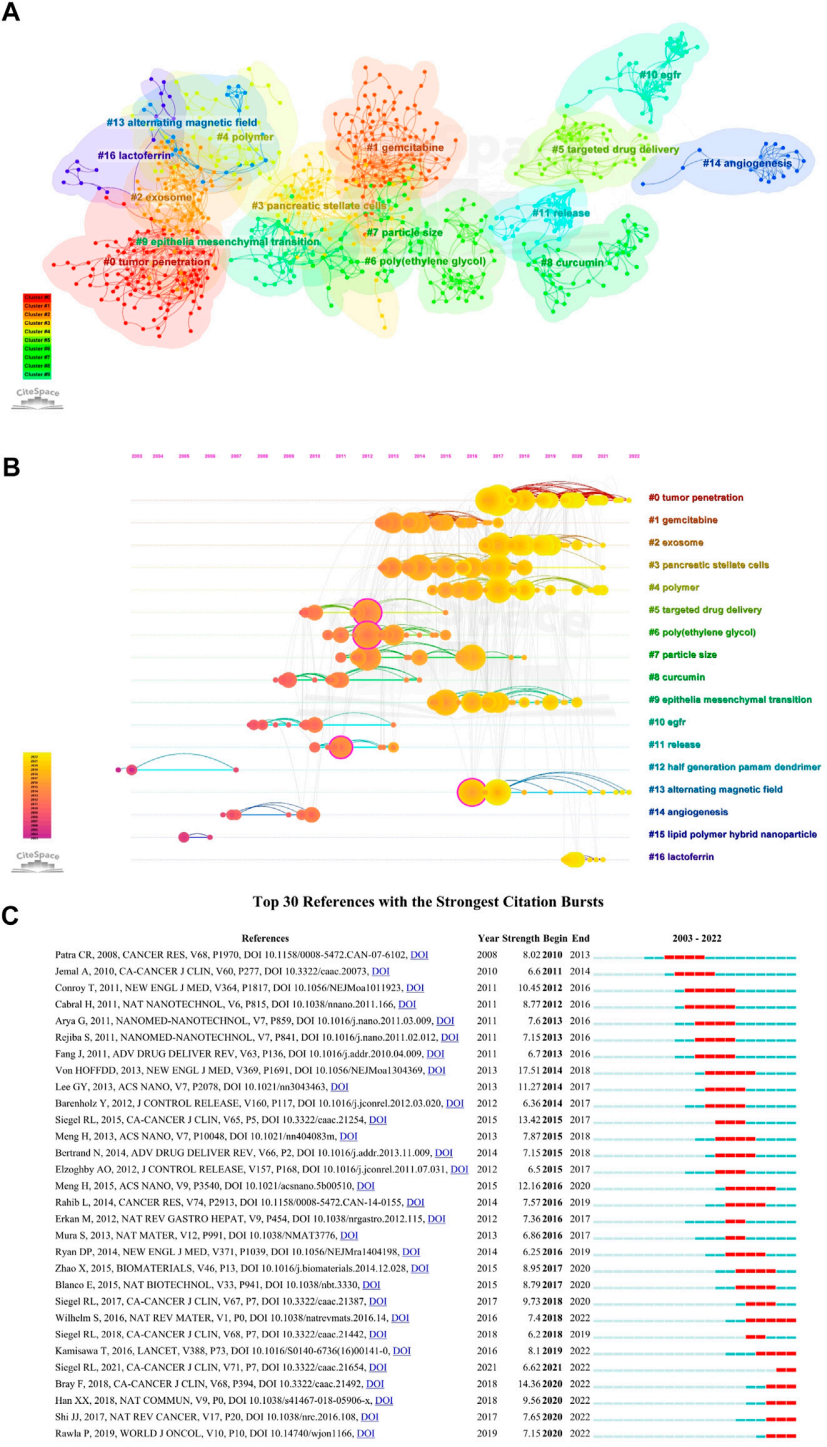
**(A)** The visualization network of co-cited references generated by Citespace. Each circle represents a reference, and circles with the same color represent a cluster with the same topic. **(B)** The timeline view map of references co-cited analysis generated by CiteSpace; This map implies the differences in the appearance time point of 17 clusters (2003–2022). The position of each circle on the horizontal axis indicates the time point of the first appearance, the size of the circle represents the total number of it was cited, and the circle on the same line represents a cluster with the same topic; The lines connecting the nodes represent co-cited relationships. **(C)** Top 30 references with the strongest citation bursts. The strength value represents the strength of citation bursts. The red bars indicate the durations of the bursts.

Table 3 summarized the top 10 highly co-cited references of NDDS for PC research. The visualization network of co-cited references by CiteSpace were shown in Figure 8A. In the visualization network of co-cited references, all nodes representing the references were clustered into 17 specific clusters with the highest K values, including “#0 tumor penetration”, “#1 gemcitabine”, “#2 exosome”, and “#3 pancreatic stellate cells”, and so on. The timeline view could help us understand the evolution track of this field (Liu et al., 2021b). Visualized timeline for these 17 clusters was further performed (Figure 8B), and we found that “half generation PAMAM dendrimer” and “lipid polymer hybrid nanoparticle” were earlier fields on NDDS for PC. In addition, we also applied CiteSpace to identify the top 30 references with the strongest citation burst. References citation burst refers to references that have been widely cited by other studies over a period of time, which meant that they have received special attention in a certain period (Min et al., 2022). As shown in Figure 8C, since 2008, the strongest citation burst came from the paper of Von HOFFDD et al. (Von Hoff et al., 2013) in 2013, followed by the article of Bray F et al. on *CA-CANCER J CLIN* in 2018 (Bray et al., 2018), and the article of Siegel RL et al. on *CA-CANCER J CLIN* in 2015 (Siegel et al., 2015).

**TABLE 3:** Top 10 highly co-cited references.

	First authors	Title	Journals	Citations
1	Von HOFF DD	Increased survival in pancreatic cancer with nab-paclitaxel plus gemcitabine	NEW ENGLAND JOURNAL OF MEDICINE	121
2	Cabral H	Accumulation of sub-100 nm polymeric micelles in poorly permeable tumours depends on size	NATURE NANOTECHNOLOGY	97
3	Burris HA	Improvements in survival and clinical benefit with gemcitabine as first-line therapy for patients with advanced pancreas cancer: a randomized trial	JOURNAL OF CLINICAL ONCOLOGY	96
4	Matsumura Y	A new concept for macromolecular therapeutics in cancer chemotherapy: mechanism of tumoritropic accumulation of proteins and the antitumor agent smancs	CANCER RESEARCH	85
5	Conroy T	FOLFIRINOX versus gemcitabine for metastatic pancreatic cancer	NEW ENGLAND JOURNAL OF MEDICINE	80
6	Peer D	Nanocarriers as an emerging platform for cancer therapy	NATURE NANOTECHNOLOGY	79
7	Meng H	Use of a lipid-coated mesoporous silica nanoparticle platform for synergistic gemcitabine and paclitaxel delivery to human pancreatic cancer in mice	ACS NANO	73
8	Olive KP	Inhibition of Hedgehog signaling enhances delivery of chemotherapy in a mouse model of pancreatic cancer	SCIENCE	67
9	Jain RK	Delivering nanomedicine to solid tumors	NATURE REVIEWS CLINICAL ONCOLOGY	59
10	Patra CR	Targeted delivery of gemcitabine to pancreatic adenocarcinoma using cetuximab as a targeting agent	CANCER RESEARCH	58

### Keywords

**FIGURE 9:**
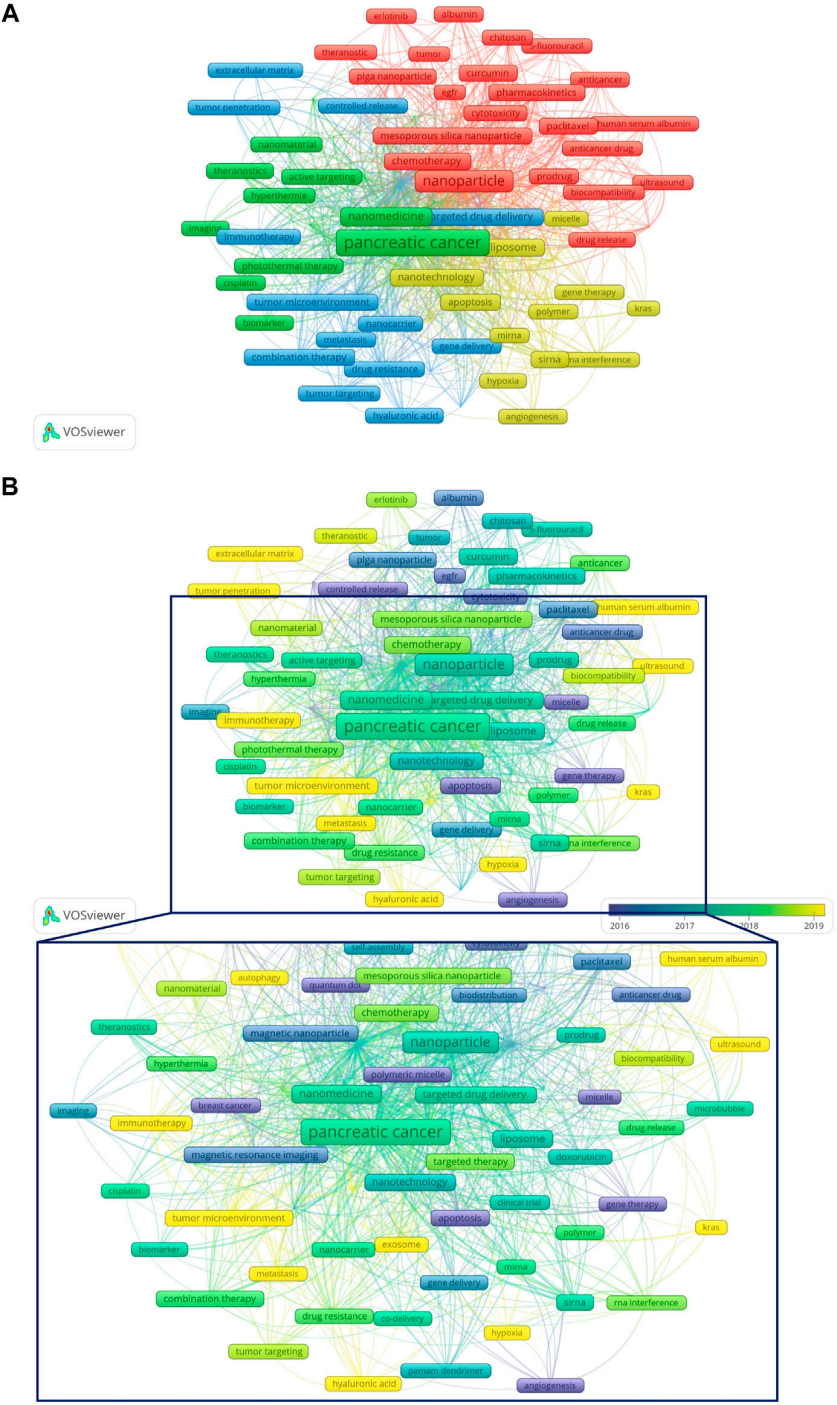
Keywords regarding NDDS for PC. **(A)** Network visualization of keywords generated by VOSviewer. **(B)** Overlay visualization of keywords generated by VOSviewer. Earlier keywords were displayed in blue, while orange represented the most recent keywords. In order to display the local area more precisely, the local area is magnified (the area enclosed by the box). Abbreviation: NDDS, Nano-drug Delivery System; PC, pancreatic cancer.

**FIGURE 10:**
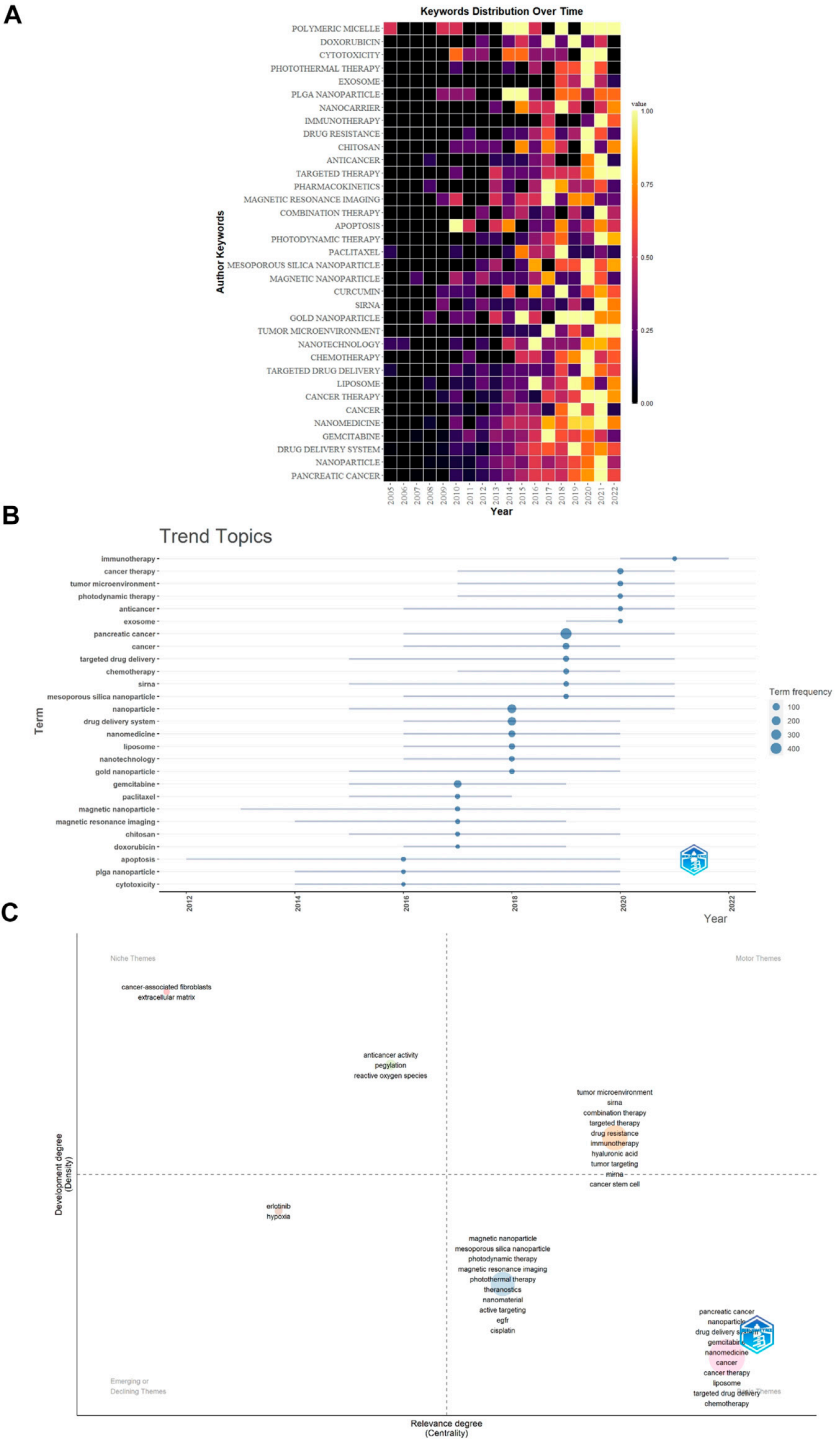
**(A)** The distribution map of 35 high-frequency keywords over time generated by R software. Each cell represented the occurrence frequency of a keyword in a year, and the corresponding value was formed after standardizing these occurrences frequencies (0–1). **(B)** Trend topics. The X-axis represents the year, while the Y-axis is the cumulate occurrences of the keywords. **(C)** The keywords thematic map generated by R-Bibliometrix. The X-axis represents the centrality indicating the importance of a theme; The Y-axis symbolizes the densityindicating the development of a theme.

Figures 9A,B show the network visualization of keywords generated by VOSviewer. Among 2713 keywords, the frequency of occurrence was set to at least 8, and finally, 82 keywords were included in the analysis. Figure 9B showed the overlay visualization of author keywords. Earlier keywords were displayed in blue, while orange represented the most recent keywords. For example, keywords such as “angiogenesis”, “EGFR”, and “controlled release” were the main topics in the early stage, and the keywords of “exosome”, “hypoxia”, “autophagy”, “tumor penetration”, and “immunotherapy” were hot topics in recent years. In addition, the distribution map of 35 high-frequency keywords over time was conducted by R software (Figure 10A), in which each cell represented the occurrence frequency of a keyword in a year, and the corresponding value was formed after standardizing these occurrences frequencies (0–1). The value of the black cell was the smallest, which represented the lowest occurrence frequency of the keyword this year, with the change of color, the value of the yellow cell was the biggest, and its corresponding keywords appeared the most frequently this year. For example, from 2002 to 2022 (as of September 8), “Magnetic resonance imaging” appeared more frequently in 2017, while in 2020 and 2021, the frequency decreased for two consecutive years.

The trend topic analysis was an important mapping tool that helped to portray the seed of trend integration rooted in the previous stream (Dai et al., 2022). The trend topics map was generated by R-Bibliometrix based on the occurrence frequency of author keywords and set word minimum frequency = 15 and the number of words per year = 6 (Figure 10B). The results showed that the duration of “apoptosis” was the longest (8 years), followed by “magnetic nanoparticle” (7 years). “Immunotherapy” began to appear in the field of NDDS for PC in 2020, and “exosome” began to appear in the field in 2019. “Immunotherapy” had the highest frequency in 2021, while “exosome”, “tumor microenvironment”, and “photodynamic therapy” had the highest frequency in 2020.

Finally, the keywords thematic map was generated by R-Bibliometrix (Figure 10C), and a total of 260 keywords were examined where a minimum cluster frequency was 6 and the number of labels for each cluster was 10. The upper right quadrant (motor theme), which was characterized by a high density and centrality, showed probably the well-developed and important themes for the structuring of the NDDS for PC research field. The cluster included “tumor microenvironment”, “siRNA”, “combination therapy”, “targeted therapy”, “drug resistance”, “immunotherapy”, “hyaluronic acid”, “tumor targeting”, “miRNA”, and “cancer stem cell”. The upper-left quadrant (niche theme) contained two clusters, cluster one included “cancer-associated fibroblasts” and “extracellular matrix”; cluster two included “anticancer activity”, “pegylation”, and “reactive oxygen species”. The cluster in the third quadrant (emerging or declining theme) was characterized by low centrality and density, which means that it was weakly developed and marginal, including “erlotinib” and “hypoxia” as the major themes. The fourth quadrant (basic themes) contained two clusters, cluster one included “pancreatic cancer”, “nanoparticle”, “drug delivery system”, “gemcitabine”, “nanomedicine”, “cancer”, “cancer therapy”, “liposome”, “targeted drug delivery”, and “chemotherapy”; cluster two included “magnetic nanoparticle”, “mesoporous silica nanoparticle”, “photodynamic therapy”, “magnetic resonance imaging”, “photothermal therapy”, “theranostics”, “nanomaterial”, “active targeting”, “EGFR”, and “cisplatin”. They concern general topics that were transversal to different research areas of the field.

## Discussion

In this study, we used CiteSpace, VOSviewer, and R-Bibliometrix to conduct a bibliometric and visual analysis of 1329 publications related to NDDS for PC published between 2003 and 2022 (as of September 8), to sort out the research status of global publications related to this field, summarize research hotspots and predict future research trends.

The change in the number of academic publications is an important indicator of the development trend of a field (Cheng et al., 2022a). Over the past 20 years, publications related to NDDS for PC have shown a significant growth trend (Figure 2). From 2002 to 2007, the number of publications related to NDDS for PC was still small (no more than 10) and the trend was unstable; From 2008 to 2011, although there were more than 10 publications per year, the trend was still unstable, and the number of publications decreased slightly in 2011; Since 2012, the number of the publications increased steadily, and the annual number of publications has exceeded 100 since 2016, although there was a slight decline in 2018. 86.3% of the papers (*n* = 1,174) were published in the past 10 years (2012–2021). These results showed that in the past 10 years, the research on NDDS for PC has begun to develop rapidly.

The number of publications in a country is an important indicator of a country’s output. Figure 3A showed that the United States, four Asian countries (China, India, Japan, and South Korea), and European countries (France, Italy, Spain, and England) almost accounted for the top 10 contributions to NDDS for PC. The United States had the highest total number of citations and H-index. These results reflected that the United States had made great contributions and established its leading position in the field of NDDS for PC. In addition to the research topic of NDDS for PC, similar results were also obtained in the bibliometric analysis of other topics such as “pancreatic cancer” (Wang and Herr, 2022), “pancreatic stellate cells” (Yang et al., 2022b), and “inhalable nanosystems” (Huang et al., 2021). As for the cooperation among countries, it could be seen from Figure 3C that among the 20 countries with the most publications, the United States and China had the closest cooperation with other countries/regions; The United States, European countries (e.g., Italy and France), and Asian countries (e.g., China, India, and Saudi Arabia) were still the most important parts of the national cooperation landscape, and extensive cooperation had been established between them, but the cooperation of other developing countries was still relatively weak, and these countries needed to further cooperate to promote the development of NDDS for PC research field worldwide.

Among the top 22 institutions with the most publications, Chinese Acad SCI, Harvard Med Sch, and Univ Sci and Technol China had BC values greater than 0.1 (0.27, 0.11, and 0.11, respectively), indicating that these institutions were at the core of international cooperation in the field of NDDS for PC. It could be seen from Figure 4 that although some institutions had cooperation with other national institutions, such as Chinese Acad SCI’s cooperation with Univ Calif Los Angeles of the United States, it mostly cooperated with Zhejiang Univ, Fudan Univ, Shanghai Jiao Tong Univ, Sun Yat Sen Univ and other domestic institutions of China, which revealed a phenomenon, that was, in the field of NDDS for PC research, there was relatively little cooperation and exchange of results between institutions in different countries, and most of the cooperation institutions were limited to the domestic. Considering that nanomedicine was more and more widely used in oncology and other medical fields, and the research was gradually in-depth, this situation has greatly hindered the development of related research fields. Therefore, it is imperative to strengthen the cooperation between international institutions and jointly promote the development of research in this field.

Among the top 5 authors who published the most papers, Couvreur P of Paris-Saclay University contributed the most papers, with the highest total and average number of citations (4,976 and 248.8, respectively); Although the total and average number of citations of the publications by Kazuoka K ranked second (3,784 and 189.2, respectively), the H-index was the highest (*n* = 19); Although Meng H’s number of publications ranked fifth, the whose total and average number of citations ranked third (1,918 and 137, respectively). As could be seen from Figure 5B, among the top 5 authors, Kataoka K carried out research in this field first, starting in 2005, with the largest annual number of publications in 2014 (*n* = 4), and the highest total citations per year in 2011 (*n* = 1,870); Among the 2 articles published by Kataoka K in 2011, the total number of citations for one article was as high as 1,727, the authors compared the accumulation and effectiveness of different sizes of long-circulating, drug-loaded polymeric micelles (with diameters of 30, 50, 70 and 100 nm) in both highly and poorly permeable tumors. All the polymer micelles penetrated highly permeable tumors in mice, but only the 30 nm micelles could penetrate poorly permeable tumors to achieve an antitumor effect, and they found that the penetration and efficacy of the larger micelles could be enhanced by using a transforming growth factor-beta inhibitor to increase the permeability of PC (Cabral et al., 2011a). Meng H carried out research in this field first, starting in 2009, with the largest annual number of articles in 2017 and 2021 (*n* = 3), and the highest total citations per year in 2009 (*n* = 687): This highly cited paper showed that polyethyleneimine (PEI) polymers could enhance the cellular uptake of mesoporous silica nanoparticles (NPs) and improved the delivery of the hydrophobic anticancer drug paclitaxel to pancreatic cancer cells (Xia et al., 2009). Couvreur P started research in this field in 2011. The author’s annual output of articles (*n* = 6) and total citations per year (*n* = 4,306) reached the highest in 2013, and one of the six articles had high scientific influence in the field of NDDS for PC research: In the review (Mura et al., 2013), the authors discussed recent advances in the design of nanoscale stimuli-responsive systems that were able to control drug biodistribution in response to specific stimuli, either exogenous (variations in temperature, magnetic field, ultrasound intensity, light or electric pulses) or endogenous (changes in pH, enzyme concentration or redox gradients). However, from 2020 to 2021, Couvreur P did not publish any research on NDDS for PC. Kataoka K made contributions to nano polymer micelles, nano gene delivery targeting PC, and nano mediated sonodynamic therapy, etc, and Meng H mainly contributed to silicasome nanocarrier delivery system of chemotherapeutic drugs for PC, nano-enabled PC immunotherapy, and the targeting strategies for the carcinogenic molecular mechanism of PC, etc, while Couvreur P made contributions to squalene-based nanosystems for controlled drug release, and stimuli-responsive nanocarriers for drug delivery, etc.

As for the author co-citation analysis, 85 authors cited at least 45 times were included. As shown in Figure 5D, the top three authors with the largest TLS were Meng H, Von Hof DD, and Maeda H. At the same time, it was worth noting that although only 7 papers on NDDS for PC were published, Cabral H from Univ Tokyo still occupied an important position in the co-citation map, which might be related to several highly cited papers they have published, especially the article published on Nature Nanotechnology in 2011 contributed as the first author, which was cited 1,727 times (Cabral et al., 2011b). This result showed that the number of publications was not the only indicator of the author’s academic influence in this field.

Analyzing the characteristics of international peer-reviewed journals is helpful to understand the current trend, which is directly reflected in helping scholars understand the important journals related to the field of NDDS for PC, and select the most appropriate published journals for their research. In this study, the 16 journals with the highest productivity were all JCR Q1 or Q2 journals, among which *JOURNAL OF CONTROLLED RELEASE*, *BIOMATERIALS*, and *INTERNATIONAL JOURNAL OF NANOMEDICINE* were the three journals with the highest productivity in the field of NDDS for PC. It could be seen from Figure 6B that since 2017, the annual publications on NDDS for PC published by *PHARMACEUTICS* increased significantly, and *PHARMACEUTICS* published the most papers among the 16 journals in 2021 and 2022 (as of September 8), which showed that the attention of this journal to the related research of NDDS for PC was increasing or the attention of scholars of this field to *PHARMACEUTICS* was increasing. The top five journals commonly co-cited were *JOURNAL OF CONTROLLED RELEASE*, *BIOMATERIALS*, *ACS NANO*, *CANCER RESEARCH*, and *ADVANCED DRUG DELIVERY REVIEWS*. Therefore, the research results related to NDDS for PC published in these journals may be easier to be cited and receive more attention. Moreover, it is necessary to pay attention to the published papers in these journals to obtain the latest progress in the field of NDDS for PC.

Figure 8C listed the top 30 references with strong citation burst. Since 2008, the strongest citation burst came from the article of Von HOFFDD et al. (Von Hoff et al., 2013) in 2013, followed by the article of Bray F et al. on *CA-CANCER J CLIN* in 2018 (Bray et al., 2018), and the article of Siegel RL et al. on *CA-CANCER J CLIN* in 2015 (Siegel et al., 2015). Considering the strength and time of burst, four articles were worthy of attention: In addition to an article by Cabral et al. (2011c), an article by Meng H et al. has maintained a continuous burst from 2016 (Meng et al., 2015), they developed a mesoporous silica NPs vector for PC, which cooperatively delivered gemcitabine/paclitaxel combinations. Cell experiments found that it could inhibit the expression of cytidine deaminase and induce oxidative stress; *In vivo* experiments showed that the growth of PC was significantly inhibited and metastasis was eliminated. Another clinical trial investigated the efficacy of FOLFIRINOX in metastatic PC (Conroy et al., 2011). In addition, Wilhelm et al. analyzed the reasons for the poor efficiency of NPs delivered to solid tumors from the perspective of tumor biology and competing organs and proposed effective strategies to address this limitation (Wilhelm et al., 2016).

The references co-citation and keyword co-occurrence analysis can help to reveal the main research directions, hot spots, and evolution process in this field (Wang et al., 2021).

Figure 8B showed the trajectory of reference clusters over time. The results indicated that the current research hotspot has shifted to “#0 tumor penetration”, “#2 exosome”, “#4 polymer”, and “#13 alternating magnetic field”.

Figure 9B showed that the keywords “exosome”, “hypoxia”, “autophagy”, “tumor penetration”, “extracellular matrix”, “tumor microenvironment”, “immunotherapy”, “ultrasound”, and “hyaluronic acid”, etc. were hot topics in recent years. In addition, we also conducted a trend topic analysis based on author keywords (Figure 10B), and the results showed that “immunotherapy” began to appear in the field of NDDS for PC in 2020, and “exosome” began to appear in the field in 2019. “Immunotherapy” had the highest frequency in 2021, while “exosome”, “tumor microenvironment”, and “photodynamic therapy” had the highest frequency in 2020. The results based on the trend topic analysis showed that in 2020 and 2021, researchers gradually paid more attention to these keywords. We also used R software to conduct a distribution map of the top 35 high-frequency keywords over time (Figure 10A), and the results showed that the two core keywords “pancreatic cancer” and “nanoparticle” had the highest frequency in 2021, and “drug delivery system” had a higher frequency in 2021, which indicated that research related to NDDS for PC was still hot; Besides, “nanomedicine”, “cancer therapy”, “nanotechnology”, “tumor microenvironment”, “siRNA”, “photodynamic therapy”, “combination therapy”, “targeted therapy”, “immunotherapy”, “cytotoxicity”, and “polymetric micelle” also had the highest frequency in 2021, and the frequency of some of these keywords fluctuated significantly: For example, “tumor microenvironment” had a relatively higher frequency in 2017 and 2021, but the frequency was lower in 2018 and 2020. Similar phenomena also occurred in “polymetric micelle”. Different software and research methods have their advantages and can complement each other. In this study, we used VOSviewer, R software, and R-Bibliometrix to analyze keywords, using keyword co-occurrence network and overlay visualization maps, trend topics map, and distribution map of high-frequency keywords over time to display the topic evolution and hotspots of keywords as broadly and objectively as possible. Keywords’ thematic map can analyze topics that may have long-term development in the future. The keywords that appear in the motor themes are important and mature. Figure 10C showed that the motor theme contained one cluster, which included “tumor microenvironment”, “siRNA”, “combination therapy”, “targeted therapy”, “drug resistance”, “immunotherapy”, “hyaluronic acid”, “tumor targeting”, “miRNA”, and “cancer stem cell”.

The above results showed that the current research hotspots of PC NDDs mainly focused on the tumor microenvironment and its molecular mechanisms, as well as topics related to tumor penetration, such as “tumor microenvironment”, “extracellular matrix”, “tumor penetration”, “hypoxia”, “exosome”, and “autophagy”; Treatment related topics, such as “immunotherapy”, “combination therapy”, “alternating magnetic field/magnetic hyperthermia”, and “ultrasound”; And gene therapy dominated by “siRNA” and “miRNA”.

An important feature of PC is the extensive deposition of extracellular matrix (ECM) components in the tumor microenvironment (TME) and the activation of cancer-associated fibroblasts (CAFs). These changes can reduce vascular patency, lead to hypoxia (Jain et al., 2014), hinder the effective delivery of drugs (Jia et al., 2021), and change the anti-tumor immune response (Hosein et al., 2020). As key participants in TME, CAFs can promote ECM deposition by producing fibrotic compounds such as collagen, hyaluronic acid, and fibronectin (Tian et al., 2019), and also have complex crosstalk with cancer and immune cells (Kota et al., 2017). In addition, CAFs can secrete chemokines, cytokines, growth factors, microRNAs (miRNAs), and extracellular vesicles to communicate with cancer cells and other TME participants to promote tumor progression (Vennin et al., 2018). Therefore, blocking the activation and proliferation of CAFs or targeting them for drug delivery is a new therapeutic strategy for PC. Study shows that the absorption of NPs by CAFs is more than 10% higher than that of tumor cells (Alhussan et al., 2021), so drugs targeting CAFs can give full play to the advantages of NDDS. Although CAFs-targeting NPs can lead to significant inactivation of CAFs, and further reduce ECM production (Feng et al., 2020a), however, blocking the activation of CAFs alone may not be effective in killing PC. Therefore, the use of nanocarriers combined with drugs that block CAFs from producing matrix and chemotherapeutic drugs (such as paclitaxel, etc.) has become a direction worthy of in-depth research (Zhao et al., 2018; Yu et al., 2020; Zang et al., 2022). In addition, targeted therapy against the over-expressed enzymes on the membrane of CAFs can also inhibit the function of CAFs, which can effectively combine with NDDS and significantly enhance drug accumulation at tumor sites (Yu et al., 2020).

The low tumor penetration dominated by TME is the core factor hindering the clinical efficacy of NDDS, mainly because of the complex arrangement and distribution of blood vessels in tumors (Jain and Stylianopoulos, 2010; Matsumoto et al., 2016), which is mainly manifested in, on the one hand, the rapid diffusion of tumor cells leads to the lack of oxygen and nutrition, which leads to vascular abnormalities and heterogeneity (Chauhan et al., 2011), and ultimately inhibits the penetration of NPs (Jain, 2003); The uneven distribution of blood vessels from the periphery to the center of the tumor further hinders the deep penetration of NPs into the tumor (Munir, 2022). On the other hand, the ECM has very narrow pores, which hinders the delivery of NPs by electrostatic interactions and steric restriction (Stylianopoulos et al., 2010; Sriraman et al., 2014). Therefore, destroying tumor ECM and complex blood vessel distribution becomes one of the main methods to improve the penetration of NPs (Munir, 2022). However, forcefully destroying the matrix barrier and the blood vessels in the tumor may lead to the imbalance of signal transduction and dependence in TME, and uncontrolled PC growth and metastasis (Chen et al., 2022). Researches show that photothermal therapy (PTT) can promote NPs to penetrate deep in sites away from vasculature exposed to the near-infrared laser (He et al., 2015; Chen et al., 2017; Yu et al., 2017), and enhance the accumulation and efficacy of chemotherapy drugs in PC (Yu et al., 2020; Zhang et al., 2021). Combined ultrasound microbubble technology can also enhance the permeability of NPs and inhibit the growth of PC (Xing et al., 2016; Xu et al., 202b). In addition, the transcellular drug transport mediated by tumor penetrating peptide iRGD has been proved to enhance the effective penetration of NPs in PC tissues (Liu et al., 2017a; Liu et al., 2017b; Ruoslahti, 2017; Hurtado de Mendoza et al., 2021), which shows the potential of iRGD as a tumor-specific enhancer. However, due to the complexity of the biological barrier, the penetration depth of NPs still needs to be determined by in-depth research. The combination of photodynamic therapy (PDT) and PTT-mediated NPs and checkpoint blocking immunotherapy (Liu et al., 2019b; Sun et al., 2021; Qiu et al., 2022a; Xu et al., 2022a; Yun et al., 2022), as well as the combination of NPs and small interacting RNA (siRNA) targeted to checkpoints (Barshidi et al., 2022; Won et al., 2022), is expected to effectively activate the immune system, cause tumor degeneration, and get rid of the technical difficulties of tumor penetration.

However, due to the abnormal vascular network in TME and other reasons, the drugs delivered by traditional nano delivery systems show reduced biocompatibility, low permeability, retention effect, and high toxicity (Zhou et al., 2020). The targeted stimulus-responsive NDDS is a very promising solution, which shows high stability, biocompatibility, enhanced permeability, reduced toxicity, and retention effect, and can improve the efficacy of delivered drugs and significantly reduce side effects (Masood, 2016; Mahato, 2017; Narayanaswamy and Torchilin, 2019). Currently, a large number of studies on NDDS targeting different stimuli-responsive elements have been carried out, including temperature (Yang et al., 2018; Lu and Ten Hagen, 2020; Zhang et al., 2022b), magnetic field (Chen et al., 2019; Sun et al., 2020), light (Sun et al., 2019; Ge et al., 2021; Feng et al., 2022), pH (Li et al., 2016a; Qu et al., 2017; Latorre et al., 2019), ATP (Lai et al., 2015; Zhang et al., 2016), enzyme (Cai et al., 2020; Deng et al., 2022b), redox-potential (Raza et al., 2018; Kumar et al., 2019), and hypoxia (Kulkarni et al., 2018; Im et al., 2019; Zhen et al., 2019; Zhu et al., 2019). However, there are still few studies on PC-targeted stimuli-responsive NDDS. It can be predicted that, unlike other solid tumors, TME of PC is more specific and complex, which is a difficult problem to overcome in the further study of targeted stimuli-responsive NDDS.

Exosomes, saucer-shaped extracellular vesicles of approximately 30–100 nm diameter that are delimited by a lipid bilayer, contain a large amount of biologically active molecules, such as lipids, enzymes, metabolites, and various non-coding RNAs (miRNAs, long noncoding RNAs, and circular RNAs) (Yu et al., 2021), and play important roles in cell-cell communications in TME (Aguilar-Cazares et al., 2022; Ghosh and Ghosh, 2022). In addition, due to the excellent biosafety, low immunogenicity, carrier properties, nanoscale penetration effect, longer half-life, and no microvascular embolism (Ghosh et al., 2020), numerous studies have shown that as a carrier of conventional chemotherapy drugs (Zhao et al., 2021; Premnath et al., 2022), targeted therapy (Deng et al., 2021; Xu et al., 2021), or mediate photodynamic therapy and immunotherapy (Jang et al., 2021), exosomes have shown potential as a new NDDS for the treatment of PC. However, the isolation and preparation of a large number of engineered exosomes is still an important test for PC treatment, which requires the support of materials science, engineering, and other disciplines; In addition, when exosomes are coupled with NPs or encapsulated with drugs, the pharmacokinetics of exosomes *in vivo* is still not completely clear, and a large number of retention effects or drug off-target phenomena may occur, affecting the efficacy; In addition, a lot of work is needed to confirm the biosafety, targeted efficacy, and adverse reactions of exosomes before clinical use.

As an extremely complex bidirectional regulatory mechanism in the development of PC, autophagy can inhibit the transformation from precancerous lesions to PC in the initial stage, and also contribute to PC progression and chemotherapy resistance (Ma et al., 2021), which is closely related to TME. Nanomaterials have been designed to inhibit the progression of PC or induce the death of PC cells by autophagy or as autophagy inhibitor carriers (Raju et al., 2018; Li et al., 2019; Thomas et al., 2020; Chen et al., 2022). However, it is also noteworthy that autophagy has also been considered an important mechanism of nanomaterial-induced toxicity (Feng et al., 2020b). Studies have reported organ damage related to nanomaterial-driven autophagy, including hepatotoxicity (Li et al., 2015; Zhang et al., 2017; Zhu et al., 2017), pulmonary toxicity (Park et al., 2015; Jiang et al., 2018b), nephrotoxicity (Lin et al., 2016; Jiang et al., 2018a), neurotoxicity (Gao et al., 2015; Shang et al., 2021), cardiovascular toxicity (Guo et al., 2016; Zhang et al., 2019b), and these organ damage processes include nanomaterial mediated autophagy triggered mitochondrial damage (Yuan et al., 2017), lysosomal dysfunction (Wang et al., 2017), endoplasmic reticulum damage (Wei et al., 2017), cytoskeleton damage (Liu et al., 2019a), Golgi body injury (Huang et al., 2018), and DNA damage (Sadhu et al., 2018), etc. Therefore, the rational use of nanomaterials to mediate autophagy in the treatment of PC still needs further research, such as the bidirectional regulation mechanism of autophagy, avoiding organ damage, and so on.

Immune checkpoint inhibitors targeting PD-1/PD-L1 have shown good efficacy in many solid tumors (Zhang et al., 202a). However, most PCs are resistant to PD-(L)1 treatment (Feng et al., 2017), and immunosuppressive TME is the intrinsic core of PC treatment resistance (Amrutkar and Gladhaug, 2017; Karamitopoulou, 2020; Zhang et al., 2022a; Sally et al., 2022). The dense fibrous matrix and deficient vascular system in PC TME can hinder the entry of effector T cells (Sahin et al., 2017; Gong et al., 2018). Moreover, the unique PC TME results in a physical barrier preventing the infiltration of chimeric antigen receptor T (CAR-T) cells. TME immune cells secrete and express molecules that suppress T cell activation, limiting CAR-T cells’ antitumor response (Bailey et al., 2016). Therefore, it can be predicted that TME will be the main mechanism that hinders the efficacy of immunotherapy for PC for a long time. However, some NDDS has shown the potential to overcome the poor immunotherapeutic efficacy caused by TME: such as NDDS to promote pyroptosis (Xiao et al., 2021; Qiu et al., 2022b), intelligent stimulus response NDDS (Bu et al., 2021; Tan et al., 2021; Wang et al., 2022c; Mao et al., 2022), NDDS with high efficiency of transcytosis (Wang et al., 2022b; Wu et al., 2022); vaccines based on NDDS (Banerjee et al., 2019; Affandi et al., 2020; Liu et al., 2021a; Tang et al., 2022); NDDS of siRNAs targeted immune (Ghasemi-Chaleshtari et al., 2020; Deng et al., 2022a; Biber et al., 2022; Li et al., 2022; Yoo et al., 2022). The development of multifunctional NDDS with diverse and complementary functions is expected to enhance the efficacy of PC immunotherapy.

Magnetic hyperthermia (MH) refers to a new therapeutic method for cancer by converting electromagnetic energy into local heat by magnetic NPs *via* hysteresis or Néel/Brownian relaxation exposed to the alternating magnetic field (AMF) (Chiu-Lam and Rinaldi, 2016; Song et al., 2020). Compared with PTT, magnetic hyperthermia has little damage to normal tissues (Tamura et al., 2022). Moreover, magnetic NPs in AMF have been proved to be able to efficiently deliver chemotherapeutic drugs and other molecular drugs, and show remarkable efficacy against various tumors including PC (Wang et al., 2015; Sanhaji et al., 2019; Zuo et al., 2021; Demin et al., 2022; Wang et al., 2022d). However, for magnetic NPs used for drug delivery, it is necessary to optimize the size and shape to achieve safe blood circulation, prolong the circulation time, and reduce the release of unsafe degradation products into the blood circulation. It is worth mentioning that stealthy design has initially shown the potential to address the above limitations (Salmaso and Caliceti, 2013; Friedl et al., 2021). The magnetic coil used for delivering the magnetic field of magnetic NPs also has technical challenges and needs to solve the precise solutions of MH combined with chemotherapy, such as duration and frequency (Palzer et al., 2021).

A recent bibliometric study showed that the combination of NDDS and ultrasound microbubbles has become a hot spot to improve tumor efficacy (Wu et al., 2021). Ultrasound microbubbles can improve membrane permeability, making therapeutic drugs easy to pass through biological barriers such as vascular endothelium and cell membrane (Meng et al., 2021; Snipstad et al., 2021). Compared with higher-frequency ultrasound, low-frequency ultrasound microbubbles are more effective in promoting the accumulation of NPs in PC tissues (Lin et al., 2018). The latest research by Meng et al. reported that the optimized NPs with virus-mimic surface topology combined with low-frequency ultrasound microbubbles significantly enhanced the permeability of the biological barrier and enhanced the distribution of NPs in solid tumors (Meng et al., 2021). In recent years, the combination of NDDS and ultrasound microbubbles has shown great potential in improving gene transfection efficiency in gene therapy (Tay and Xu, 2017; Wang et al., 2022a; Kida et al., 2022), and improving the efficacy of chemotherapy drugs on a variety of tumors including PC (Gao et al., 2019; Lee et al., 2020; Liu et al., 2020; Yang et al., 2022a; Ngamcherdtrakul et al., 2022; Schoen et al., 2022). However, it should be noted that the size and concentration of microbubbles and the total amount of gas contained in the liquid are important factors affecting NDDS mediated by ultrasound microbubbles (Kida et al., 2022).

In addition to the use of chemotherapy drugs and immunotherapy, drugs such as siRNA and miRNA targeting different targets are also important cancer therapies. Various methods have been proven to be able to achieve targeted inhibition of specific miRNAs (Sun et al., 2015; Liang et al., 2016; Van Roosbroeck et al., 2017; De Cola et al., 2018). However, susceptibility to degradation by nucleases, low intracellular absorption rate due to an inherent negative charge and hydrophilic structure, and potential off-target effects limit the application of miRNAs (Kara et al., 2022). MiRNA nanocarriers based on polymers, inorganic materials, and lipids have demonstrated their potential in the treatment of PC (Arora et al., 2014; Gurbuz and Ozpolat, 2019; Gokita et al., 2020; Wu et al., 2020; Borchardt et al., 2022). However, it is important to note that therapeutic miRNAs can also accumulate in healthy tissues due to interstitial fluid pressure and shearing stress promoting NPs extravasation, which may lead to their degradation (Zhang et al., 2019a), and cause toxic and other adverse reactions (Kara et al., 2022). Effective delivery and cell uptake of siRNA are major challenges in therapeutic applications (Wang et al., 2016; Arnold et al., 2017; Tan et al., 2022). PEGylated ligand-targeted liposomes or micelles avoid nonspecific clearance of siRNA by the reticuloendothelial system and protect siRNA from the degradation of nuclease (Charbe et al., 2020), which makes it possible to address these major obstacles. Although several studies have shown that the NDDS can effectively deliver siRNA and synergize with chemotherapeutic drugs to inhibit the growth of PC and the expression of related genes (Li et al., 2016b; Strand et al., 2019; Wang et al., 2020; Jung et al., 2021; Luo et al., 2021), however, the biosafety and biodegradability of siRNA delivery vehicles, the targeting specificity and endosomal escape capability of nanocarriers to tumor tissues, toxicity and immune stimulation need to be addressed by further studies (Aghamiri et al., 2021; Huang and Xiao, 2022).

Dual-map overlays showed that NDDS for PC research mainly focuses on molecular, biology, immunology, and physics, materials, chemistry (Figure 7). Fortunately, these researches related to NDDS for PC has reflected the effective penetration of multiple disciplines and the result of deepening cooperation, which is also the development trend of various disciplines at present, especially in nanomaterials and medicine.

## Limitations

There were several limitations. First, this study only included the publications from 2003 to 2022 (as of September 8), and it was possible to miss some important and landmark studies before 2002; Secondly, because the literature of 2022 was incomplete and some unpublished hot spots may not be included, and some results of this study may change after being included in the data for the whole year of 2022, so what is necessary to carry out follow-up research to evaluate the results more objectively; Finally, although we used various software to analyze the content of countries, institutions, authors, keywords, and references, the analysis still could not provide a comprehensive overview of NDDS for PC-related literature. However, we believe that the current literature-based bibliometric studies may, to the greatest extent, allow scholars to understand the research hotspots and development trends of NDDS for PC.

## Conclusion

In the past 20 years, the number of publications related to NDDS for PC has increased, indicating that the interest of scholars in this field is increasing. The United States is absolutely in the leading position in the field of NDDS for PC research. In terms of publication volume and global institutional cooperation, Chinese Acad SCI has made the greatest contribution in this field. Professors Couvreur P and Kazuoka K made great achievements in this field. *JOURNAL OF CONTROLLED RELEASE* is at the core of the publishing of NDDS for PC research. The topics related to the TME such as “tumor microenvironment”, “tumor penetration”, “hypoxia”, “exosome”, and “autophagy”, PC treatment-related topics such as “immunotherapy”, “combination therapy”, “alternating magnetic field/magnetic hyperthermia”, and “ultrasound”, and gene therapy dominated by “siRNA” and “miRNA” are the research hotspots and trends in the field of NDDS for PC.

## Data Availability

The original contributions presented in the study are included in the article/Supplementary Material, further inquiries can be directed to the corresponding author.
